# Comparison of procedural outcomes in patients undergoing catheter vs surgical ablation for atrial fibrillation and heart failure with reduced ejection fraction

**DOI:** 10.1002/joa3.12451

**Published:** 2020-11-23

**Authors:** Rajkumar Doshi, Ashish Kumar, Mariam Shariff, Devina Adalja, Krunalkumar Patel, Kirtenkumar Patel, Rupak Desai, Nageshwara Gullapalli, Saraschandra Vallabhajosyula

**Affiliations:** ^1^ Department of Internal Medicine University of Nevada Reno School of Medicine Reno NV USA; ^2^ Department of Critical Care Medicine St John’s Medical College Hospital Bengaluru India; ^3^ Department of Medicine GMERS Gotri Medical College Vadodara India; ^4^ Department of Medicine St Mary Medical Center Langhorn PA USA; ^5^ Department of Cardiology North Shore University Hospital Manhasset NY USA; ^6^ Department of Cardiology Atlanta VA Medical Center Decatur GA USA; ^7^ Section of Interventional Cardiology Division of Cardiovascular Medicine Department of Medicine Emory University School of Medicine Atlanta GA USA

**Keywords:** atrial fibrillation, catheter ablation, HFrEF, surgical ablation

## Abstract

**Background:**

There is a lack of research comparing procedural outcomes of surgical ablation (SA) and catheter ablation (CA) among patients with heart failure with reduced ejection fraction (HFrEF) and atrial fibrillation (AF). The main objective was to compare the short‐term procedural outcomes of SA and CA in patients with HFrEF.

**Methods:**

We used the national inpatient sample to identify hospitalizations over 18 years with HFrEF hospitalization and AF, and undergoing SA and CA from 2016 to 2017. Furthermore, the clinical outcomes of SA vs CA in AF stratified as nonparoxysmal and paroxysmal were analyzed.

**Results:**

A total of 1,770 HFrEF hospitalizations with AF who underwent SA and 1,620 HFrEF hospitalizations with AF who underwent CA were included in the analysis. Hospitalizations with CA had higher baseline comorbidities. The in‐hospital mortality among HFrEF with AF undergoing SA as compared with CA was similar (2.8% vs 1.9%, respectively, adjusted P‐value 0.09). Hospitalizations with SA had a significantly longer length of hospital stay, a higher percentage of postprocedural, and cardiac complications. In HFrEF hospitalizations with nonparoxysmal AF, SA as compared with CA was associated with a higher percentage of in‐hospital mortality (2.4% vs 1%, adjusted *P*‐value <.05), a longer length of stay, a higher cost of treatment, and a higher percentage of cardiac complications.

**Conclusion:**

CA is associated with lower in‐hospital adverse procedural outcomes as compared with SA among HFrEF hospitalizations with AF. Further research with freedom from AF as one of the outcome is needed between two groups for HFrEF.

## Graphical abstract

Catheter ablation is associated with lower in‐hospital procedural outcomes as compared to surgical ablation among heart failure with reduced ejection fraction hospitalizations. The recurrence of atrial fibrillation, long‐term safety, efficacy, and quality of life associated with surgical and catheter ablation remain to be determined in this population.

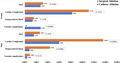

## 1 INTRODUCTION

Atrial fibrillation (AF) is the most common cardiac rhythm dysfunction affecting ~5.3 million people across the United States.^1^ The varied treatment strategies of AF extend across a wide spectrum, including medical therapy (rate or rhythm control), catheter ablation (CA), and surgical ablation (SA). Traditionally, the standard of care for drug‐refractory AF was SA.^2^ However, recent advancements with the application of CA for the management of refractory AF have increased significantly owing to its low complexity and adverse events.^3^ Hence, CA is approved for drug‐refractory AF by European and American guidelines.^4^, ^5^ Few randomized clinical trials published comparing SA vs CA have highlighted CA to have less consistent maintenance of sinus rhythm postprocedure.^6^, ^7^, ^8^ However, these clinical trials were bound by the limitations in terms of strict patient selection and limited patient population. Additionally, these trials also excluded patients with an ejection fraction below 45%.^6^, ^7^, ^8^

There is a lack of research comparing procedural outcomes of SA and CA among heart failure patients with reduced ejection fraction (HFrEF) or systolic heart failure and AF. The evidence is indispensable as CA is performed more frequently among subjects with HFrEF.^9^ Therefore, we assessed short‐term procedural outcomes comparing SA and CA in patients with HFrEF and AF. We have also expanded our research by dividing our patient population into paroxysmal and nonparoxysmal AF.

## 2 METHODS

### 2.1 Data source and study population

The study cohort was derived from the National Inpatient Sample (NIS). The NIS is created and maintained by the Agency for Healthcare Research and Quality, for the Healthcare Cost and Utilization Project (HCUP).^10^ The NIS is the largest all‐payer inpatient health care database in the United States, yielding national estimates of hospital inpatient stays. Unweighted, the NIS contains data from more than 8 million hospital stays each year and weighted, the NIS estimates more than 40 million hospitalizations from >4000 hospitals nationally. International classification of disease, Tenth Revision, Clinical Modification (ICD‐10‐CM) diagnosis code I50.2 was used to identify hospitalizations with HFrEF hospitalization from January 1st, 2016 to December 31st, 2017. We excluded admissions before 2015 since it utilized ICD‐9‐CM codes. Moreover, ICD‐10‐CM includes specific codes for the diagnosis and procedures included in the present analysis. The NIS variables provided by the sponsor were used to identify baseline characteristics and demographics. We also included long‐term use of aspirin, anticoagulation, and antiplatelet agents using ICD‐10‐CM codes. The length of prescription and compliance with these medications was unknown, given the nature of the database. The NIS database has been explained in detail in the past.^11^, ^12^ Since the NIS data includes de‐identified administrative data with prior ethical committee approval, no additional ethical committee approval was deemed necessary for the present analysis.

**FIGURE 1:**
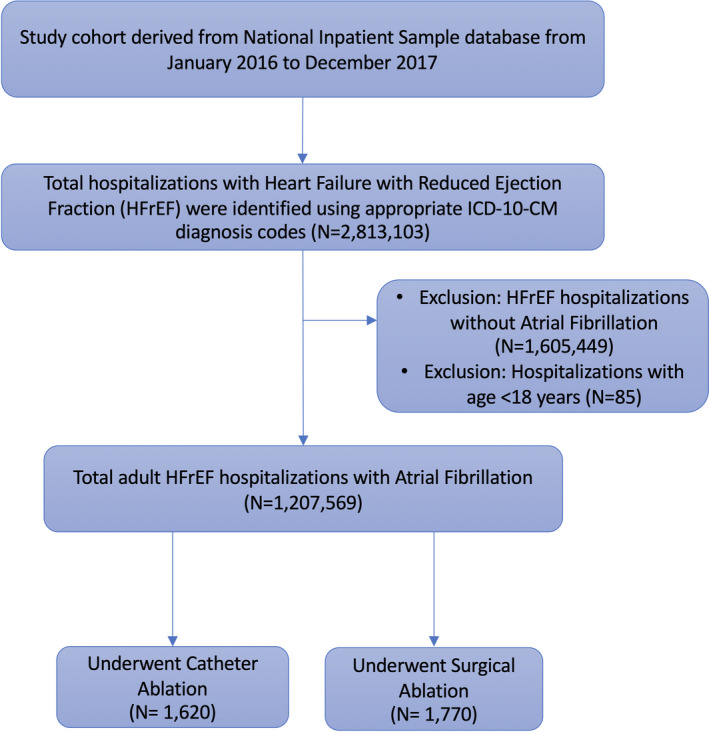
Flow chart for the selection of the hospitalizations

First, we identified hospitalizations with HFrEF using ICD‐10‐CM diagnosis code I50.2. I50.x is a validated ICD‐10‐CM code for heart failure, used in a previous study.^13^ Among these HFrEF admissions, hospitalizations over 18 years with AFs were identified using ICD‐10‐CM diagnostic codes I48.0, I48.1, I48.2, I48.91.^14^ Alongside this, we excluded hospitalizations who did not have AF and age below 18 years. Among the HFrEF with AF patients undergoing CA and SA were identified using ICD‐10‐CM procedure codes (surgical ablation: 02560ZZ, 02570ZZ, 025K0ZZ, 025L0ZZ, 02B60ZZ, 02B70ZZ, 02BK0ZZ, 02BL0ZZ, 02T80ZZ; catheter ablation: 02563ZZ, 02573ZZ, 025K3ZZ, 025L3ZZ, 02B63ZZ, 02B73ZZ, 02BK3ZZ, 02BL3ZZ). (Figure 1) The ICD‐10‐CM codes used to discern comorbidities and complications among identified hospitalizations are provided in the Supplementary Table S1. The AF hospitalizations were further stratified based on paroxysmal and nonparoxysmal AF, and the respective outcomes with CA compared with SA were investigated. Additionally, outcomes were stratified based on gender and race. The primary outcome was in‐hospital all‐cause mortality for all the analyses. The secondary outcomes included length of stay, cost of hospitalization, disposition status postdischarge, vascular complications, postoperative shock, and cardiac complications. The ICD‐10‐CM codes utilized for these outcomes are detailed in Supplementary Table S1.

### 2.2 Statistical analysis

All statistical analyses were performed using SAS version 9.4. The statistical analysis was performed strictly in accordance with the Agency for Healthcare Research and Quality and expert consensus recommendations.^15^ The analysis accounted for the survey design (SURVEYMEANS and SURVEYFREQ), clustering (HOSP_NIS), and weights (DISCWT). This is in accordance with the methodological standard required by the sponsor.^15^ All tests were two‐sided and a level of significance was set to a *P*‐value <.05. Continuous variables were expressed as mean (standard deviations [SD]) or median (interquartile range [IQR]) depending on the data distribution. Categorical data were expressed as percentages. Intergroup and intragroup comparisons of continuous variables were evaluated using the Wilcoxon rank‐sum test, and categorical variables were evaluated using the Chi‐square test. The total charge for each hospital stay was converted to cost estimates using the group average all‐payer in‐hospital cost and charge information from the detailed reports by hospitals to the Centres for Medicare and Medicaid Services. The final cost was calculated by multiplying the total cost with the cost‐to‐charge ratio provided by the sponsor. Multivariable logistic regression analysis was used to account for confounder associated with outcomes. The following variables were used for regression analysis age, gender, race, primary payer, comorbidities, medication use, hospital region, and hospital setting. The comorbidities used for regression analysis were history of hypertension, diabetes, smoking, obesity, alcohol abuse, drug abuse, chronic lung disease, chronic liver disease, chronic renal failure, coronary artery disease, valvular heart disease, and obstructive sleep apnea.

## 3 RESULTS

A total of 1,770 HFrEF hospitalizations with AF who underwent SA and 1,620 HFrEF hospitalizations with AF who underwent CA were included in the analysis. Table 1 delineates the demographic and baseline characteristics of hospitalizations who underwent SA in contrast to CA in HFrEF hospitalization with AF. The mean age of the hospitalizations undergoing SA and CA were similar (~66 years). Females underwent SA more frequently, contrary to CA (35.6% vs 22.5%, *P* < .001). Blacks underwent CA more frequently (15.7% vs 7.6%, P value < .001), while whites underwent SA more frequently (75.1% vs 68.8%, P value < .001). Medicare was the main payor, covering over 60% of the total procedures. Hospitalizations with private insurance underwent SA more frequently as opposed to CA (27.4% vs 19.7%, *P* < .001), while in hospitalizations with Medicare (63.6% vs 61.6%, *P* value < .001) and Medicaid (11.7% vs 8.2%, *P* value < .001) underwent CA more commonly. Hospitalizations undergoing CA had higher percentage of diabetes (29.3% vs 21.7%), history of smoking (40.7% vs 33.9%, *P* value < .001), and history of drug abuse (3.4% vs 0.8%, *P* value < .001), chronic lung history (26.2% vs 17.5%, *P* value < .001), and chronic renal failure (29.9% vs 17.8%, *P* value < .001) contrasting to SA. Furthermore, hospitalizations for CA had a higher percentage of long‐term aspirin use and antithrombotic/antiplatelet therapy (6.2% vs 1.7%, *P* < .001). However, no statistically significant difference was present in the long‐term use of anticoagulation therapy. Most of the procedures were performed in the Southern part of America compared to other regions of the United States. Most of these procedures were performed in the urban teaching hospital with no difference in the frequency of SA and CA. The in‐hospital mortality among HFrEF with AF undergoing SA as compared with CA was similar (2.8% vs 1.9%, respectively, adjusted *P*‐value .09) (Table 2). However, the hospitalizations for SA had a lower percentage of discharge to home in contrast to CA (33% vs 65.9%, adjusted *P* < .001). Hospitalizations for SA had a significantly longer length of hospital stay (11[7‐17] vs 5[3‐10], adjusted *P* value < .001), a higher percentage of postprocedural (3.4% vs 0.9%, adjusted *P* value < .001), and cardiac complications (12.7% vs 6.5%, adjusted *P* value < .001). The vascular complication was similar among CA as compared with SA (0.9% vs 0.6%, adjusted P‐value 0.14) hospitalizations. CA was significantly cheaper as compared to SA among HFrEF hospitalizations with AF ($34,513 vs $57,416, adjusted *P*‐value < .001).

**TABLE 1:** Demographics and baseline characteristics of HFrEF hospitalizations with atrial fibrillation undergoing surgical vs catheter ablation

Variable name	With surgical ablation N = 1770 (52.2%)	With catheter ablation N = 1620 (47.8%)	*P* value
Age, years (mean ± SD)	66.6 ± 10.9	66.2 ± 11.3	.64
Gender			<0.001
Female	35.6%	22.5%	
Male	77.5%	64.4%	
Race			<0.001
White	75.1%	68.8%	
Black	7.6%	15.7%	
Other	17.2%	15.4%	
Primary Payer			<0.001
Medicare	61.6%	63.6%	
Medicaid	8.2%	11.7%	
Private Insurance	27.4%	19.7%	
Other	2.8%	4.9%	
Comorbidities			
Hypertension	44.3%	43.5%	.62
Diabetes	21.7%	29.3%	<.001
Smoking	33.9%	40.7%	<.001
Obesity	17.5%	18.5%	.44
Alcohol Abuse	3.1%	4%	.15
Drug Abuse	0.8%	3.4%	<.001
Chronic Lung Disease	17.5%	26.2%	<.001
Chronic Liver Disease	1.9%	1.8%	.79
Chronic Renal Failure	17.8%	29.9%	<.001
Coronary Artery Disease	57.9%	56.8%	.51
Valvular Heart Disease	0%	0.3%	.01
Obstructive Sleep Apnea	17.2%	17.6%	.78
Medication Use			
Long‐term use of anticoagulation	37%	40.1%	.06
Long‐term use of aspirin	16.1%	21.3%	.001
Long‐term use of antithrombotics/antiplatelets	1.7%	6.2%	<.001
Hospital Level Variables			
Hospital Region			<0.001
Northeast	17.8%	22.2%	
Midwest	24.3%	20.7%	
South	38.7%	40.1%	
West	19.2%	16.9%	
Hospital Setting			0.15
Rural	1.7%	1.2%	
Urban, nonteaching	14.1%	12.3%	
Urban, teaching	84.2%	86.4%	

**TABLE 2:** Primary and secondary outcomes in HFrEF hospitalizations with atrial fibrillation undergoing surgical vs catheter ablation

Outcomes	With surgical ablation (N = 1770)	With catheter ablation (N = 1620)	Unadjusted *P* value	Adjusted *P* value
In‐hospital Mortality	2.8%	1.9%	.06	.09
Discharge to Home	33%	65.9%	<.001	<.001
Length of Stay, in days (Median, IQR)	11 (7‐17)	5 (3‐10)	<.001	<.001
Cost, in $ (Median, IQR)	57,416 (42,523‐86,917)	34,513 (23,136‐48,271)	<.001	<.001
Vascular Complication	0.6%	0.9%	.21	.14
Postprocedural Shock	3.4%	0.9%	<.001	<.001
Cardiac Complication	12.7%	6.5%	<.001	<.001

### 3.1 Subanalysis of hospitalizations with nonparoxysmal AF

Table 3 highlights demographic and baseline characteristics of HFrEF hospitalizations with nonparoxysmal AF stratified based on SA or CA procedure. Following multivariate logistic regression, HFrEF hospitalizations with nonparoxysmal AF, SA as opposed to CA was associated with a higher percentage of in‐hospital mortality (2.4% vs 1%, adjusted *P* value < .05), a longer length of stay (11 vs 5, adjusted *P* value < .001), a higher cost of treatment (58,462 [43,443‐84,415] 33,246 [22,362‐44,644], adjusted *P* value < .001), and a higher percentage of cardiac complications (10.2% vs 6.8%, adjusted *P* value < .05). The number of hospital admissions disposed to home were significantly higher with CA (67.2% vs 35.1%, adjusted *P* value < .05 (Table 4).

**TABLE 3:** Demographics and baseline characteristics of HFrEF hospitalizations with nonparoxysmal atrial fibrillation undergoing surgical vs catheter ablation

Variable name	With surgical ablation N = 1025 (51.6%)	With catheter ablation N = 960 (48.4%)	*P* value
Age (mean ± SD)	67 ± 10.7	66.7 ± 11	.77
Gender			<.001
Female	35.6%	22.4%	
Male	64.4%	77.6%	
Race			<0.001
White	75.6%	67.2%	
Black	7.8%	17.7%	
Other	16.6%	15.1%	
Primary Payer			<0.001
Medicare	62.4%	63.5%	
Medicaid	7.3%	11.5%	
Private Insurance	27.8%	19.8%	
Other	2.4%	5.2%	
Comorbidities			
Hypertension	45.4%	45.8%	.83
Diabetes	24.9%	29.2%	.03
Smoking	33.2%	39.6%	.003
Obesity	19.5%	19.3%	.89
Alcohol Abuse	2.9%	4.2%	.13
Drug Abuse	1.5%	4.2%	<.001
Chronic Lung Disease	18.5%	26%	<.001
Chronic Liver Disease	1.9%	1.6%	.51
Chronic Renal Failure	17.1%	30.7%	<.001
Coronary Artery Disease	57.1%	54.7%	.28
Valvular Heart Disease	0	0.5%	.02
Obstructive Sleep Apnea	19%	19.8%	.67
Medication Use			
Long‐term use of anticoagulation	38.5%	39.6%	.63
Long‐term use of aspirin	12.7%	22.9%	<.001
Long‐term use of antithrombotics/antiplatelets	1.5%	4.7%	<.001
Hospital Level Variables			
Hospital Region			0.27
Northeast	20%	22.9%	
Midwest	21.9%	22.9%	
South	38%	36.5%	
West	20%	17.7%	
Hospital Setting			0.007
Rural	0.5%	1.6%	
Urban, nonteaching	14.6%	11.5%	
Urban, teaching	84.9%	86.9%	

**TABLE 4:** Primary and secondary outcomes in HFrEF hospitalizations with nonparoxysmal atrial Fibrillation undergoing surgical vs catheter ablation

Outcomes	With surgical ablation (N = 1025)	With catheter ablation (N = 960)	Unadjusted *P* value	Adjusted *P* value
In‐hospital Mortality	2.4%	1%	.02	.006
Discharge to Home	35.1%	67.2%	<.001	<.001
Length of Stay, in days (Median, IQR)	11	5	<.001	<.001
Cost, in $ (Median, IQR)	58,462 (43 443‐84 415)	33,246 (22 362‐44 644)	<.001	<.001
Vascular Complication	0.9%	1%	.88	.87
Postprocedural Shock	2.9%	0%	<.001	.96
Cardiac Complication	10.2%	6.8%	.005	.007

### 3.2 Subanalysis of hospitalizations with paroxysmal AF

Table 5 represents demographics and baseline characteristics of HFrEF hospitalizations with paroxysmal AF stratified based on SA or CA procedure performed. Analogous to hospitalizations with nonparoxysmal AF, following multivariate logistic regression analysis in HFrEF hospitalizations with paroxysmal AF, SA compared with CA were associated with a longer length of stay (11 vs 6, adjusted *P* value <.001), higher cost of treatment (56,795[40,291‐92,313] vs 36,188 [23,639‐56,471], adjusted *P* value < .001), and greater percentage of cardiac complications (16.1% vs 6.1%, adjusted *P* value < .001). The in‐hospital mortality (3.4% vs 3%, adjusted *P* value = .22), vascular complications (0% vs 0.8%, adjusted P value = 0.98), and postoperative shock (4% vs 2.3%, adjusted *P* value = 0.52) were similar. The number of hospital admissions disposed to home were significantly higher with CA (64.1% vs 30.2%, adjusted *P* value < .001). (Table 6).

**TABLE 5:** Demographics and baseline characteristics of HFrEF hospitalizations with paroxysmal atrial fibrillation undergoing surgical vs catheter ablation

Variable name	With surgical ablation N = 745 (53%)	With catheter ablation N = 660 (46.9%)	*P* value
Age (mean ± SD)	66.1 ± 11.1	65.5 ± 11.7	.69
Gender			<.001
Female	35.6%	22.7%	
Male	64.4%	77.3%	
Race			.002
White	74.5%	71.2%	
Black	7.4%	12.9%	
Other	18.1%	15.9%	
Primary Payer			.007
Medicare	60.4%	63.6%	
Medicaid	9.4%	12.1%	
Private Insurance	26.8%	19.7%	
Other	3.4%	4.5%	
Comorbidities			
Hypertension	42.9%	40.1%	.28
Diabetes	17.4%	29.5%	<.001
Smoking	34.9%	42.4%	.003
Obesity	14.8%	17.4%	.17
Alcohol Abuse	3.4%	3.8%	.66
Drug Abuse	0	2.3%	<.001
Chronic Lung Disease	16.1%	26.5%	<.001
Chronic Liver Disease	2%	2.3%	.73
Chronic Renal Failure	18.8%	28.8%	<.001
Coronary Artery Disease	59.1%	59.8%	.76
Valvular Heart Disease	0	0	
Obstructive Sleep Apnea	14.8%	14.4%	.84
Medication Use			
Long‐term use of anticoagulation	34.9%	40.9%	.02
Long‐term use of aspirin	20.8%	18.9%	.38
Long‐term use of antithrombotics/antiplatelets	2%	8.3%	<.001
Hospital Level Variables			
Hospital Region			<.001
Northeast	14.8%	21.2%	
Midwest	27.5%	17.4%	
South	39.6%	45.4%	
West	18.1%	15.9%	
Hospital Setting 0.003			
Rural	3.4%	0.8%	
Urban, nonteaching	13.4%	13.6%	
Urban, teaching	83.2%	85.6%	

**TABLE 6:** Primary and secondary outcomes in HFrEF hospitalizations with paroxysmal atrial fibrillation undergoing surgical vs catheter ablation

Outcomes	With Surgical Ablation (N = 745)	With Catheter Ablation (N = 660)	Unadjusted *P* value	Adjusted *P* value
In‐hospital Mortality	3.4%	3%	.75	.22
Discharge to Home	30.2%	64.1%	<.001	<.001
Length of Stay, in days (Median, IQR)	11	6	<.001	<.001
Cost, in $ (Median, IQR)	56,795 (40,291‐92,313)	36,188 (23,639‐56,471)	<.001	<.001
Vascular Complication	0%	0.8%	.02	.98
Postprocedural Shock	4%	2.3%	.06	.52
Cardiac Complication	16.1%	6.1%	<.001	<.001

### 3.3 Outcomes stratified by gender and race

**FIGURE 2:**
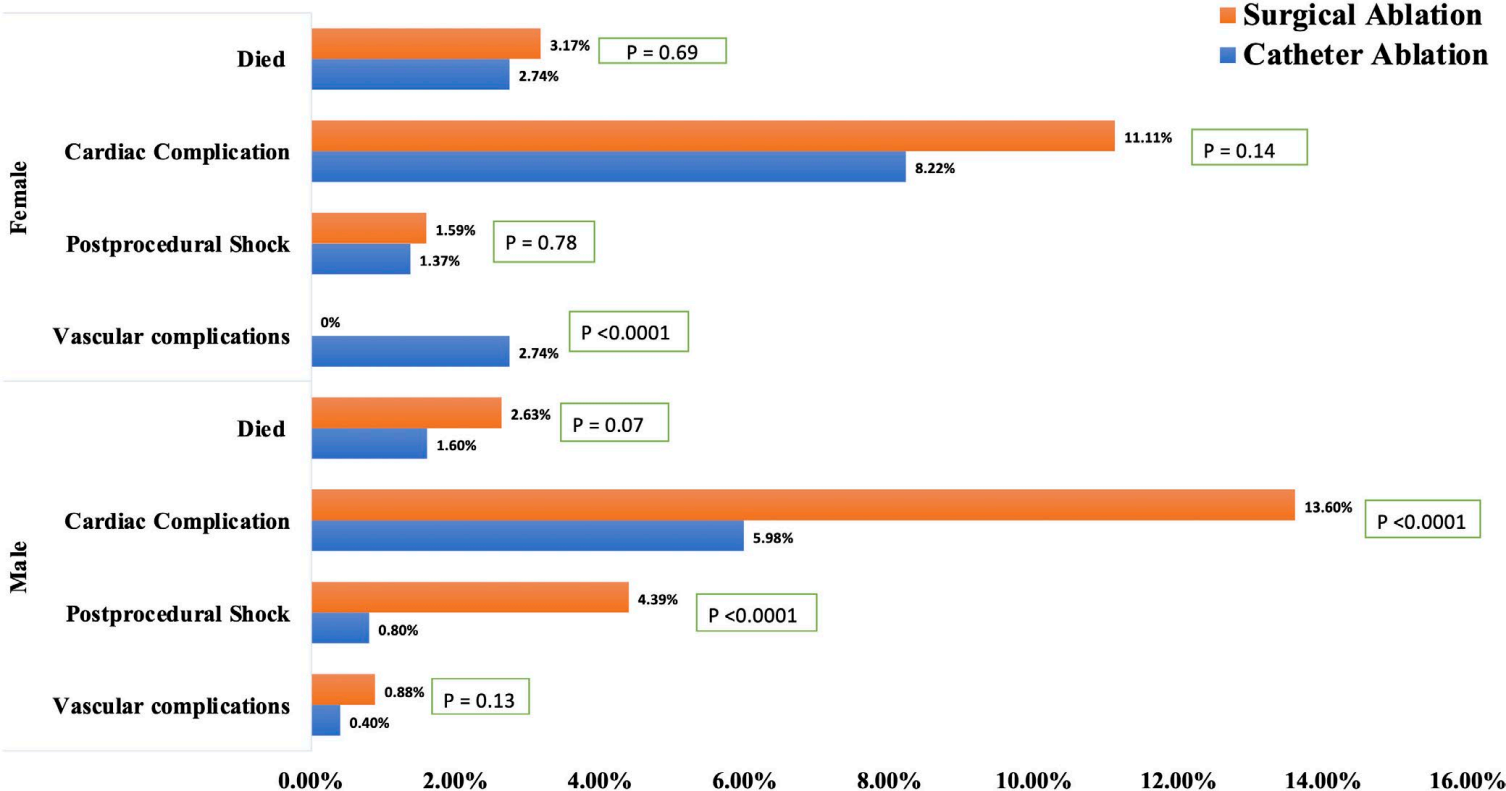
Differences in the clinical outcomes forhospitalizations with atrial fibrillation and heart failure with reduced ejection fraction: Stratified by gender and type of the procedure

**FIGURE 3:**
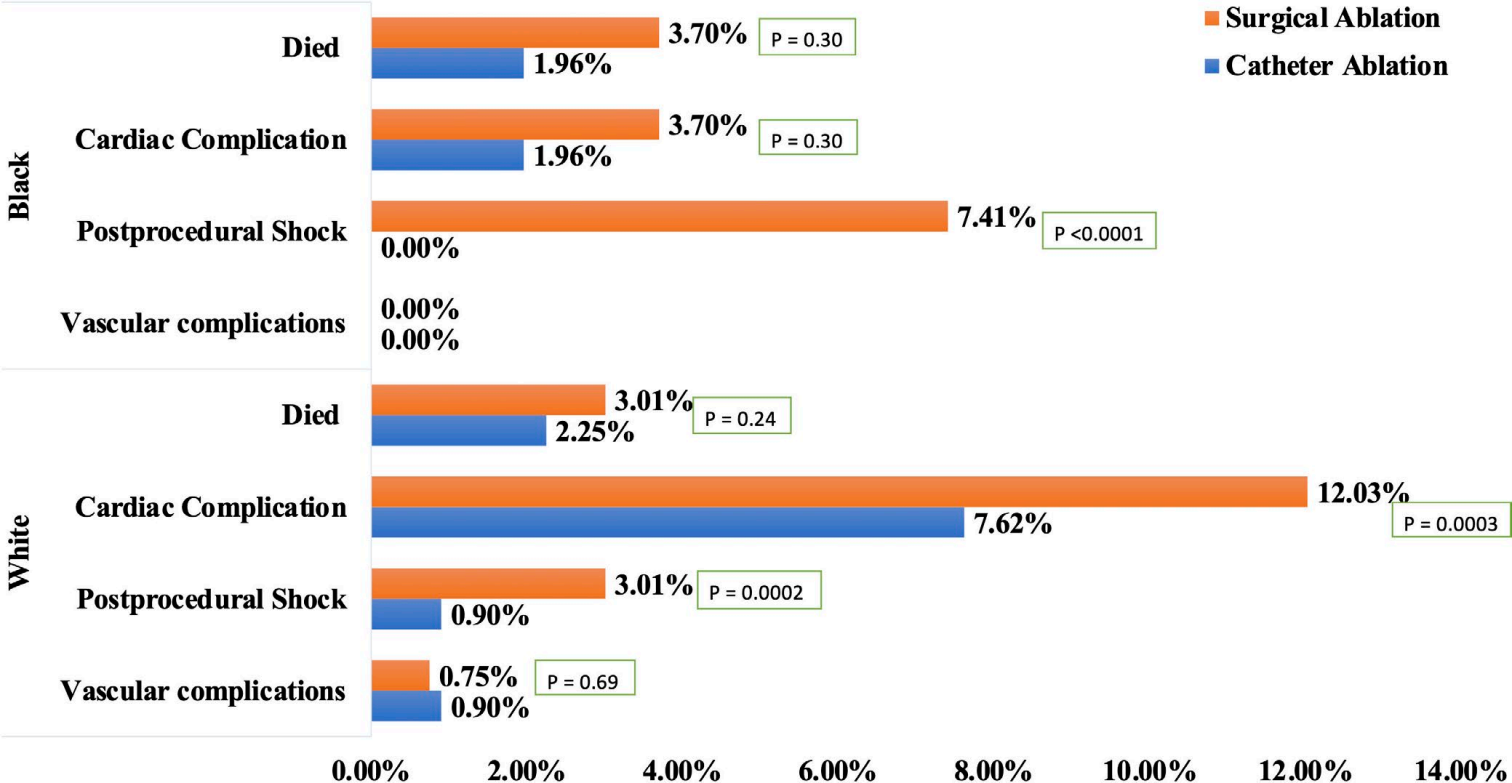
Differences in the clinical outcomes forhospitalizations with atrial fibrillation and heart failure with reduced ejection fraction: Stratified by race and type of the procedure

Figures 2 and 3 summarizes stratification of procedural outcomes among HFrEF hospitalizations with AF undergoing SA or CA, based on gender and race. Males and females, both, had lower rates of complications with CA as compared to SA. (Figure 2) Whites and Blacks, both, had lower rates of complications with CA vs SA. (Figure 3).

## 4 DISCUSSION

To the best of our knowledge, this is the first study comparing procedural outcomes of SA and CA among hospitalizations with HFrEF hospitalizations with AF. A greater percentage of females underwent SA compared with males. Although the subjects undergoing CA had higher baseline comorbidities, there was no difference in in‐hospital mortality between SA and CA. However, a significantly lower periprocedural cardiac complication, shorter length of stay, and lower cost of hospitalizations were noticed with CA compared to SA. To add on, hospitalizations with CA were disposed to home oftentimes as compared to SA. These disparities persisted in gender and race stratified analysis. In our subanalysis of hospitalizations with paroxysmal AF, there was no difference in in‐hospital mortality while, lower rates of cardiac complications, shorter length of hospitalizations, and lower cost with CA were observed. Although in another subanalysis of hospitalizations with nonparoxysmal AF, we found significantly lower in‐hospital mortality even after adjustment in addition to lower rates of cardiac complications, shorter length of hospitalizations, and lower cost with CA.

Ablation therapy using SA or CA is recommended for patients’ refractory to medical therapy. Former randomized controlled trials and meta‐analyses demonstrated better symptomatic relief from AF following SA compared with CA.^6^, ^16^, ^17^ However, SA is not employed because of its complexity with a steep learning curve, and its association with higher procedure‐related adverse outcomes.^16^ Alternatively, CA is an alternate option that has slightly lower rates of freedom from AF in contradistinction to SA, however, it is easy to learn and associated with lower procedure‐related adverse events.^16^ Additionally, randomized controlled trials studying SA opposed to CA among patients with AF included patients with prior failed CA making the success of subsequent CA unlikely.^6^, ^17^ Also, the trials excluded patients with ejection fraction <45%, and lower ejection fraction makes surgical ablation unfavorable.^18^ Despite higher baseline comorbidities among the CA group, we noticed fewer adverse effects in the CA group, with similar mortality outcomes in contrast with the SA group.

Vascular complications were similar among both groups. However, CA can be revamped with the use of ultrasound‐guided access minimizing the vascular outcomes. The higher rates of periprocedural outcomes (postprocedural shock and cardiovascular complications) with SA as compared with CA are likely mechanical and associated with injury to the adjacent structures during the procedure. The increased occurrence of periprocedural complications in SA as compared with CA has been highlighted in former meta‐analyses.^16^, ^19^ SA requires general anesthesia, which has its adverse effects. These adverse events reflect the longer length of stay and a higher cost of hospitalizations as seen in this study. Besides, these adverse events might lead to worse short‐term and long‐term quality of life. Former randomized controlled trials and meta‐analysis demonstrated SA as the preferred option for previously failed ablation attempts with AF recurrence, as they are associated with better AF‐free survival.^16^ Nonetheless, CA was associated with less in‐hospital outcomes in this study, including both subgroup analysis. Additionally, prior meta‐analyses reported better outcomes with CA as compared with medical therapy.^20^ Hence, considering the higher baseline comorbidities among heart failure patients, we suggest that CA should be the preferred first line procedure for the management of treatment‐refractory AF, especially in patients with nonparoxysmal AF and HFrEF hospitalization.

Tremendous advancement has been noted in CA techniques. Cryoballoon pulmonary vein isolation has reported promising results in the treatment of non‐paroxysmal AF.^21^ The use of radiofrequency balloon catheter and pulsed‐field ablation has also shown to improve outcomes post‐CA.^22^, ^23^ Improved imaging modality such as the use of delayed enhancement cardiac magnetic resonance can detect uncommon anatomy and fibrosis before ablation and hence can help in the better selection of patients, who will possibly benefit from the use of CA.^24^ Furthermore, second generation laser balloon can help improve tissue contact and visibility as reported by the MERLIN registry.^25^

This study has several limitations that must be taken into consideration. First, we do not have an important clinical outcome, restoration of sinus rhythm/AF recurrence, which is reported by most randomized clinical trials. This outcome along with rates of readmissions for AF should be analyzed in future, to conclude on the long‐term efficacy of catheter ablation. Second, we do not have information on the antiarrhythmic agent which was administered before and after the treatment. Third, the NIS data lack information pertaining to ejection fraction of individual patients. Hence the effect of ejection fraction on clinical adverse outcomes with SA and CA cannot be determined. Fourth, we utilized ICD‐10‐CM codes for the identification of the procedures and the disease. This might lead to a reduced number of procedures compared to the previous year which utilized ICD‐9‐CM codes which were not so specific for CA and SA.^26^

In conclusion, CA is associated with lower in‐hospital adverse procedure‐related outcomes compared with SA among HFrEF hospitalizations. The long‐term safety, efficacy, and quality of life associated with SA and CA remain to be determined in this population. A future randomized controlled trial comparing clinical outcomes and long‐term safety and efficacy in both, paroxysmal and nonparoxysmal, patients are warranted to expand the knowledgebase.

## CONFLICT OF INTEREST

The authors declare no conflict of interest.

## Supporting information

Table S1
