# Supplementary material for: Comparison of procedural outcomes in patients undergoing catheter vs surgical ablation for atrial fibrillation and heart failure with reduced ejection fraction
Source: J Arrhythm. 2020 Nov 23;37(1):60–9. doi: 10.1002/joa3.12451 (PMC7896461; doi:10.1002/joa3.12451)
Supplement: Supplementary file 1 — Table S1 [file JOA3-37-60-s001.docx]

**Supplementary Table 1: ICD-10-CM codes included in the study**

| ICD-10-CM codes | |
| --- | --- |
| Heart Failure with Reduced Ejection Fraction | I50.2 |
| Atrial Fibrillation | I48.0, I48.1, I48.2, I48.91 |
| Paroxysmal Afib | I48.0 |
| Persistence Afib | I48.1 |
| Chronic Afib | I48.2 |
| Surgical Ablation | 02560ZZ, 02570ZZ, 025K0ZZ, 025L0ZZ, 02B60ZZ, 02B70ZZ, 02BK0ZZ, 02BL0ZZ, 02T80ZZ |
| Catheter Ablation | 02563ZZ, 02573ZZ, 025K3ZZ, 025L3ZZ, 02B63ZZ, 02B73ZZ, 02BK3ZZ, 02BL3ZZ |
| Smoking | F17.210, Z87.891 |
| Coronary Artery Disease | I25 |
| Obstructive Sleep Apnea | G47.33 |
| Long term use of anticoagulation | Z79.01 |
| Long term use of aspirin | Z79.82 |
| Long term use of antithrombotics/antiplatelets | Z79.02 |
| Postprocedural Shock | T81.1 |
| Cardiac Complications | I97.710, I97.790, I97.88, I97.89, I31.2, I31.4, I31.3, 0W9C30Z, 0W9C3ZZ, 0W9D30Z, 0W9D3ZX, 0W9D3ZZ, 0W9D40Z, 0W9D4ZX , 0W9D4ZZ, 02CN0ZZ, 02CN3ZZ , 02CN4ZZ, 02NN0ZZ, 02NN3ZZ, 02NN4ZZ, 0W9D00Z, 0W9D0ZX, 0W9D0ZZ, 0WCD0ZZ, 0WCD3ZZ, 0WCD4ZZ |
| Vascular Complications | T81.7, I97.410, I97.610, I97.630, S15.009A, S15.309A, S15,209A, S15.8XXA, S09.0XXA, S15.9XXA, S25.00XA, S25.109A, S25.20XA, S25.309A, S25.409A, S25.509A, S25.809A, S25.90XA, S35.00XA, S35.10XA, S35.299A, S35.219A, S35.229A, S35.239A, S35.299A, S35.339A, S35.349A, S35.319A, S35.329A, S35.8X9A, S35.403A, S35.406A, S35.406A, S35.513A, S35.516A, S35.533A, S35.536A, S35.59XA, S35.8X9A, S35.90XA, S45.809A, S45.009A, S45.209A, S45.109A, S55.109A, S65.109A, S55.009A, S65.009A, S65.209A, S65.309A, S65.409A, S65.509A, S65.809A, S55.809A, S65.809A, S45.909A, S55.909A, S65.909A, S75.009A, S75.109A, S75.209A, S85.309A, S85.409A, S85.009A, S85.509A, S85.109A, S85.139A, S85.209A, S85.169A, S85.809A, S95.109A, S75.809A, S85.809A, S95.809A, S75.909A, S85.909A, S95.902A, T14.90XA |
